# Curcumin Ameliorates Furazolidone-Induced DNA Damage and Apoptosis in Human Hepatocyte L02 Cells by Inhibiting ROS Production and Mitochondrial Pathway

**DOI:** 10.3390/molecules21081061

**Published:** 2016-08-22

**Authors:** Chongshan Dai, Daowen Li, Lijing Gong, Xilong Xiao, Shusheng Tang

**Affiliations:** 1 College of Veterinary Medicine, China Agricultural University, 2 Yuanmingyuan West Road, Beijing 100193, China; daichongshan@163.com (C.D.); lidaowen123.fff@163.com (D.L.); 2 Sport Science Research Center, Beijing Sport University, 48 Xinxi Road, Haidian District, Beijing 100084, China; argun@126.com

**Keywords:** curcumin, furazolidone, oxidative stress, DNA damage, mitochondrial pathway

## Abstract

Furazolidone (FZD), a synthetic nitrofuran derivative, has been widely used as an antibacterial and antiprotozoal agent. Recently, the potential toxicity of FZD has raised concerns, but its mechanism is still unclear. This study aimed to investigate the protective effect of curcumin on FZD-induced cytotoxicity and the underlying mechanism in human hepatocyte L02 cells. The results showed that curcumin pre-treatment significantly ameliorated FZD-induced oxidative stress, characterized by decreased reactive oxygen species (ROS) and malondialdehyde formation, and increased superoxide dismutase, catalase activities and glutathione contents. In addition, curcumin pre-treatment significantly ameliorated the loss of mitochondrial membrane potential, the activations of caspase-9 and -3, and apoptosis caused by FZD. Alkaline comet assay showed that curcumin markedly reduced FZD-induced DNA damage in a dose-dependent manner. Curcumin pre-treatment consistently and markedly down-regulated the mRNA expression levels of p53, Bax, caspase-9 and -3 and up-regulated the mRNA expression level of Bcl-2. Taken together, these results reveal that curcumin protects against FZD-induced DNA damage and apoptosis by inhibiting oxidative stress and mitochondrial pathway. Our study indicated that curcumin may be a promising combiner with FZD to reduce FZD-related toxicity in clinical applications.

## 1. Introduction

Furazolidone (FZD), a synthetic nitrofuran drug, has been used to treat the infections caused by bacteria and protozoa in human and animals [1,2]. Recent literatures report that FZD has novel applications in treating leukemia [3] or leishmaniasis [4]. In particular, FZD is usually used as a low-cost and effective drug to treat infections caused by *Helicobacter pylori* in human in developing countries, including China [5]. However, FZD is limited in the clinic due to its potential side effects, such as genotoxicity, hepatotoxicity, and carcinogenicity [5,6,7]. A pooled-data analysis reported that FZD-based regimens achieved low eradication rates for *Helicobacter pylori* infections at the current dosage regimen, but the incidence of severe side effects was observed when the dose was increased [8]. As a result, development of agents against FZD-related adverse effects is very urgent and it is a crucial strategy for optimizing potential antimicrobial activity and clinical usage of FZD.

A previous study showed that oxidative stress may play a critical role in FZD-induced cytotoxicity and genotoxicity in human hepatoma (HepG2) cells [9]. Using the pig model the liver was suggested as the primary target organ of FZD metabolism; in addition, its metabolites 3-amino-2-oxazolidinone could accumulate in the liver [10]. The in vitro studies showed that FZD at concentrations of 4–10 µg/mL could increase the frequency of sister chromatid exchanges (SCE) in human lymphocytes and increased SCE was detected when mice were exposed to FZD at a dose of 30 mg/kg [11]. Reactive oxygen species (ROS) are mainly generated by the mitochondria [12,13]. The mitochondrion is the major mediator of oxidative stress and it is considered as the most vulnerable target in the FZD-induced cytotoxicity process [9,13]. Very recently, Deng et al. demonstrated that the mitochondrial pathway and phosphatidylinositol-3-kinase (PI3K)/Akt pathway played the critical roles in FZD-induced apoptotic cell death in HepG2 cells [14].

Curcumin, a natural polyphenol found in the spice turmeric, has many biological functions, such as anti-inflammatory, anti-oxidative, anti-carcinogenic and immuno-regulatory abilities [15]. Many studies have demonstrated that curcumin could protect against DNA damage and oxidative stress caused by some drugs or environmental mutagens, including arsenic [16], acrylamide [17] and cisplatin [18], via scavenging ROS and improving the overall anti-oxidative ability. Curcumin administration showed immense therapeutic effects against *Helicobacter pylori* infection in mice and reduced the gastric damage due to infection [19]. In addition, curcumin also showed anti-parasitic potential, including trypanocidal and leishmanicidal activity, in several in vitro and in vivo models [20,21]. Human clinical trials showed that healthy human volunteers orally administered 500 mg of curcumin per day for 7 days showed significantly decreased levels of serum lipid peroxide, a biomarker of oxidative stress [22]. Curcumin combination with some antibiotics and chemotherapy agents showed better therapeutic effect for infections and cancer compared to either one alone, which has raised wide interest in clinical practice [23,24,25]. Thus far, however, the potential preventive role of curcumin against FZD-induced adverse effects has not been investigated. Therefore, the present study investigated the protective role of curcumin on FZD-induced cytotoxicity and DNA damage using hepatocyte L02 cells, with the aim of providing a promising combination of FZD with curcumin regarding the in vitro toxicology aspect.

## 2. Results

### 2.1. Curcumin Attenuates FZD Induced Cytotoxicity in L02 Cells

**Figure 1:**
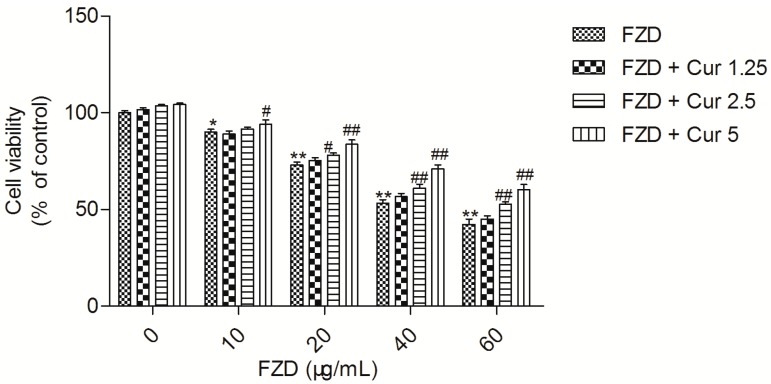
Curcumin protects against FZD induced cytotoxicity in L02 cells. Values were presented as mean ± SD, from five independent experiments. * *p* < 0.05, ** *p* < 0.01, compared to the negative control group (0.2% DMSO); ^#^ *p* < 0.05, ^##^ *p* < 0.01, compared to the FZD alone group. FZD, furazolidone.

FZD treatment for 24 h reduced the cell viability of L02 cells in a dose-dependent manner. Compared to the negative control group (0.2% DMSO), FZD treatment at 10, 20, 40 and 60 μg/mL for 24 h significantly decreased the cell viabilities to 89.4% (*p* < 0.05), 73.2% (*p* < 0.01), 53.4% (*p* < 0.01) and 42.6% (*p* < 0.01), respectively (Figure 1).

However, curcumin pre-treatment, especially at the final concentrations of 2.5 and 5 μM, markedly attenuated FZD induced cytotoxicity, compared to the FZD alone treatment groups (Figure 1). There was no significant change in cell viability in curcumin alone treatment (at 1.25, 2.5 and 5 μM, respectively) groups (Figure 1).

### 2.2. Curcumin Suppresses FZD Induced Oxidative Stress in L02 Cells

**Figure 2:**
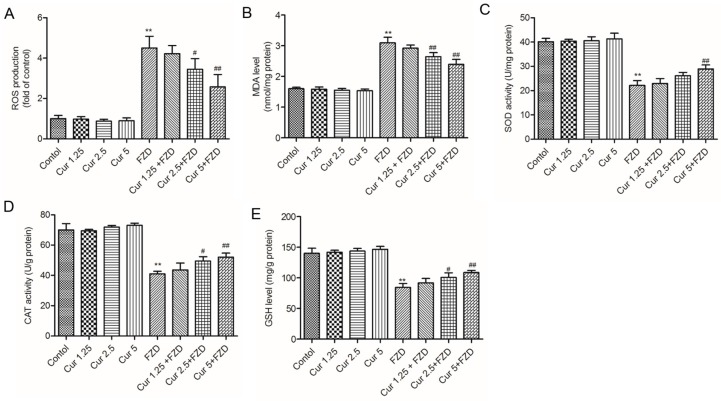
Curcumin protects against FZD induced oxidative stress in L02 cells. (**A**) Intracellular ROS levels were detected using the ROS sensitive dye 2,7-dichlorofluorescein diacetate staining; (**B**–**E**) The effects of curcumin pretreatment at 1.25, 2.5 and 5 μM on the cellular MDA level, SOD, CAT activities and GSH level. Values were presented as mean ± SD, from three independent experiments. ** *p* <0.01, compared to the negative control group; ^#^ *p* < 0.05, ^##^ *p* < 0.01, compared to the FZD alone group.

To investigate the protective effect of curcumin, the intracellular ROS and the levels of superoxide dismutase (SOD), catalase (CAT), glutathione (GSH) and malondialdehyde (MDA) were measured. As shown in Figure 2, compared to the negative control group, FZD treatment at 40 μg/mL for 24 h significantly increased the production of intracellular ROS to approximate 4.5-fold (*p* < 0.01) (Figure 2A). Meanwhile, after FZD treatment, MDA level increased to 3.1 nmol/mg protein (equal to 194% of control), and the SOD, CAT activities and GSH levels decreased to 21.3 U/mg protein, 41.1 U/mg protein and 84.5 mg/g protein (equal to 56.2%, 58.7% and 61.4% of control, respectively, all *p* < 0.01), compared to the control group (Figure 2B–E). Moreover, curcumin pre-treatment, especially at 5 μM could effectively inhibit the ROS production, decrease the levels of MDA, and increase the activities of SOD and CAT and GSH levels (all *p* < 0.01), respectively, compared to the FZD alone group (Figure 2).

### 2.3. Curcumin Protects Against FZD Induced the Loss Of Mitochondrial Membrane Potential (Δψ_m_) in L02 Cells

**Figure 3:**
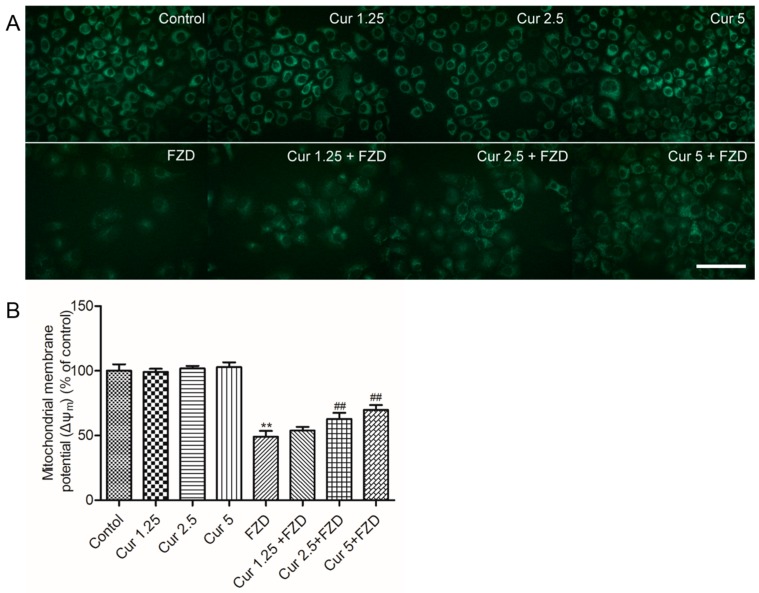
Curcumin reduces FZD induced the loss of mitochondrial membrane potential (Δψ_m_) in L02 cells. (**A**) The changes of Δψ_m_ were observed under a fluorescence microscope after stained with 10 µg/mL Rhodamine 123. Bar = 50 µm; (**B**) The average fluorescent intensities from for five independent microscopic fields were analyzed using Image Pro Plus 5.0 software (National Institute of Mental Health, Bethesda, MD, USA). The reduction of Rhodamine 123 fluorescence indicated loss of mitochondrial membrane potential. ** *p* < 0.01, compared to the negative control group; ^##^ *p* < 0.01, compared to the FZD alone treatment group.

As shown in Figure 3, FZD treatment at 40 μg/mL for 24 h significantly decreased Δψ_m_ to 49.6% (*p* < 0.01), compared to the negative control group. Curcumin treatment at 1.25, 2.5 and 5 μM had no effect on the changes of Δψ_m_, but significantly reduced the loss of Δψ_m_ caused by FZD in a dose-dependent manner (increased to 53.8%, 62.6%, 69.8%, respectively), compared to FZD alone group (Figure 3B).

### 2.4. Curcumin Inhibits FZD Induced the Activations of Caspase-9 and -3

**Figure 4:**
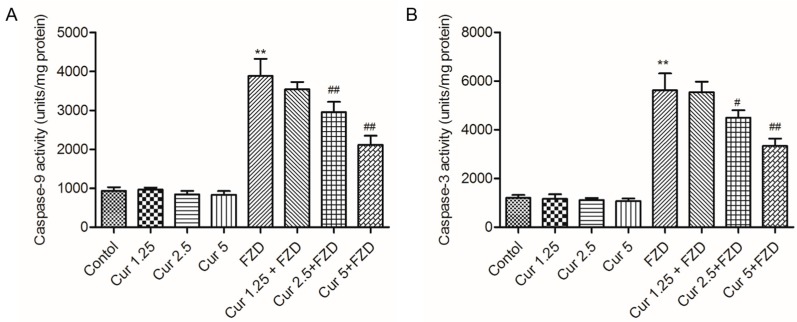
Curcumin treatment attenuated the increases of caspase-9 and -3 activities caused by FZD exposure. L02 cells were pretreated with curcumin at 1.25, 2.5 and 5 μM for 2 h, followed to wash with PBS twice, then exposed with or without FZD at 40 μg/mL for additional 24 h, caspase-9 (**A**) and -3 (**B**) activities were measured, respectively. Values were presented as mean ± SD, from three independent experiments. ** *p* < 0.01, compared to the negative control group; ^#^ *p* < 0.05, ^##^ *p* < 0.01, compared to the FZD alone treatment group.

As shown in Figure 4, FZD treatment of L02 cells at 40 μg/mL for 24 h significantly increased the activities of caspase-9 and -3 to 3884.3 and 5623.4 units/mg protein (4.2- and 4.7-fold of control), respectively, compared to the negative control group.

Curcumin pre-treatment at 2.5 and 5 μM significantly deceased the caspase-9 activity to 2951.2 and 2112.5 units/mg protein (equal to 3.2 and 2.3 folds of control) (Figure 4A), and decreased the caspase-3 activity to 4498.6 and 3334.1 units/mg protein (Figure 4B), respectively (all *p* < 0.05 or 0.01). There was no marked change of caspase-9 and -3 activities in curcumin alone treatment groups (Figure 4A,B)

### 2.5. Curcumin Reduces FZD Induced Apoptosis in L02 Cells 

**Figure 5:**
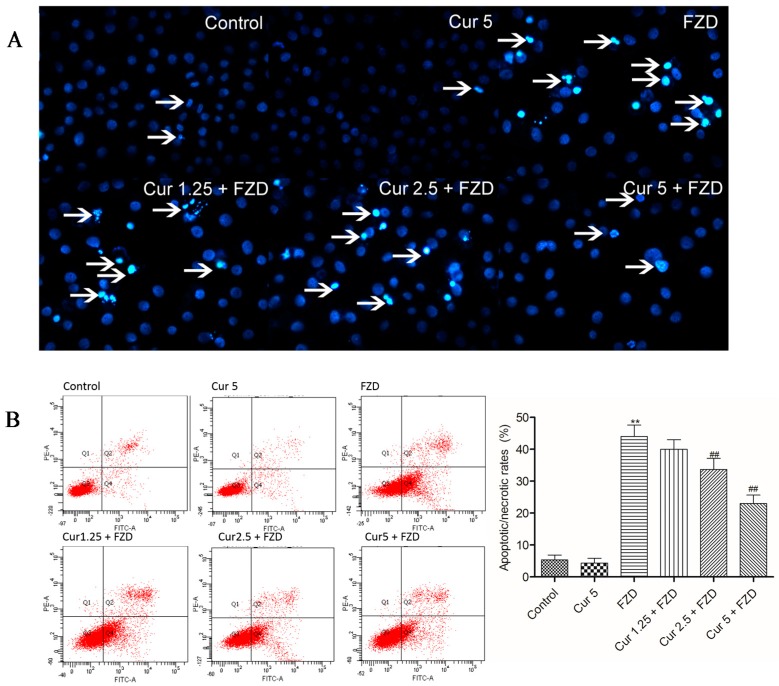
Curcumin treatment reduces FZD induced the apoptosis in L02 cells. L02 cells were pretreated with curcumin at 1.25, 2.5 and 5 μM for 2 h, followed to wash with PBS twice and expose with FZD at 40 μg/mL for additional 24 h. (**A**) Nuclear morphology of L02 cell was observed and photographed by fluorescence microscopy following Hoechst 33342 staining. The white arrows indicated apoptotic cells; (**B**) Cellular apoptosis were quantified using flow cytometry following annexin V-FITV/PI staining. Q1 necrosis cells, Q2 later apoptotic cells, Q3 survival cells, Q4 early apoptotic cells. Values were presented as mean ± SD, from three independent experiments. ** *p* < 0.01, compared to the negative control group; ^##^ *p* < 0.01, compared to the FZD alone treatment group.

After FZD treatment, L02 cells displayed condensed and fragmented chromatin and bright blue nuclei, which are indicative of apoptosis (Figure 5A). Furthermore, the apoptosis ratios were investigated by flow cytometry. Compared to the negative control group, FZD treatment increased the apoptotic rates to 43.7% (*p* < 0.01). Curcumin pretreatment at 2.5 and 5 μM significantly attenuated FZD induced nuclear condensation and fragmentation, and the apoptotic rates decreased to 33.2% and 22.6% (both *p* < 0.01) (Figure 5B), respectively, compared to the FZD treatment group. The nuclear morphology and apoptotic rate had no marked change in the curcumin alone treatment groups (Figure 5), compared to the negative control group.

### 2.6. Curcumin Reduces FZD Induced DNA Damage in L02 Cells

**Figure 6:**
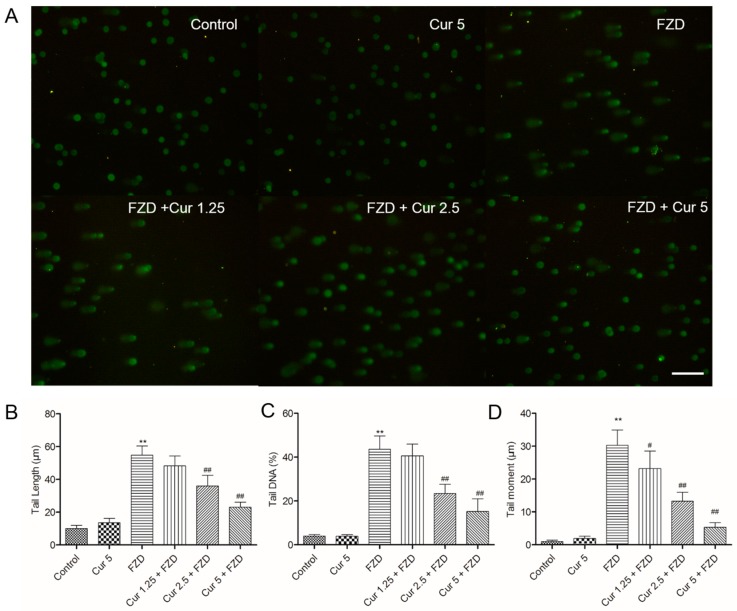
Curcumin treatment reduces FZD induced DNA injury in L02 cells. L02 cells were pretreated with curcumin at 1.25, 2.5 and 5 μM for 2 h, followed to wash with PBS twice and treat with or without FZD at 40 μg/mL for additional 3h, alkaline comet assay was carried out to evaluate the DNA injury. (**A**) The typical DNA comet images were showed. Bar = 100 μm (**B**–**D**) represented the analysis results of tail length, tail DNA (%) and Tail moment (μm), respectively. Values were presented as mean ± SD from at least 100 cells. ** *p* < 0.01, compared to the negative control group; ^#^ *p* < 0.05, ^##^ *p* < 0.01, compared to the FZD alone group.

FZD exposure at 40 μg/mL for 3 h caused L02 cells the marked DNA damage, as indicated by the greater migration of DNA fragments on the agarose gel (Figure 6A). The tail length and tail DNA (%) were both significant higher (both *p* < 0.01), compared with the negative control group (Figure 6B,C). The tail moment significantly increased to 30.2 μm, exceeding over 29 folds (Figure 6D). The pretreatment with 1.25, 2.5 and 5 μM prevented FZD-induced DNA damage in a dose-dependent manner, the tail moment decreased to 23.2, 13.5 and 5.4 μm (all *p* < 0.05 or 0.01) (Figure 6D), respectively, as well as the decreases of tail length and tail DNA (%). Curcumin treatment at 5 μM did not cause any significant increases in tail length, tail DNA (%) and tail moment, compared to the negative control group.

### 2.7. Curcumin Regulates the mRNA Expression Levels of Apoptosis Related Factors 

**Figure 7:**
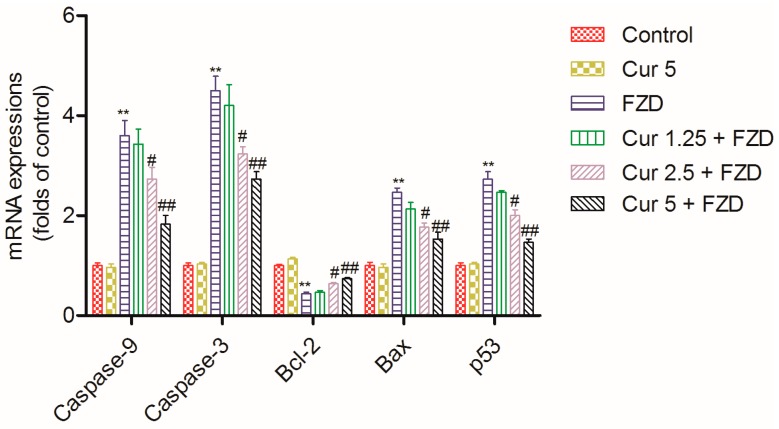
Curcumin treatment ameliorates the mRNA expression levels of apoptosis related factor caused by FZD exposure. Values were presented as mean ± SD, from three independent experiments. ** *p* < 0.01, compared to the negative control group; ^#^ *p* < 0.05, ^##^ *p* < 0.01, compared to the FZD alone treatment group.

FZD treatment at 40 μg/mL for 24 h significantly increased the mRNA expression levels of caspase-9, caspase-3, Bax and p53 by 3.6-, 4.5-, 2.4- and 2.7-fold (all *p* < 0.01), and decreased the mRNA expression level of Bcl-2 by 0.43-fold (*p* < 0.01) (Figure 7), compared to the negative control group. However, curcumin pretreatment, especially at 2.5 and 5 μM, significantly ameliorated the expression levels of these genes, i.e., caspase-9 mRNA expression level decreased 2.7- and 1.8-fold, caspase-3 mRNA expression level decreased by 3.2- and 2.6-fold, Bax mRNA expression level decreased 1.8- and 1.5-fold, p53 mRNA expression level decreased 2.0- and 1.5-fold, and Bcl-2 mRNA expression level increased 0.64- and 0.76-fold (all *p* < 0.05 or 0.01) (Figure 7), compared to the FZD alone treatment group. In the curcumin control group, the expression levels of caspase-9, caspase-3, Bcl-2, Bax and p53 showed no marked change, compared to the negative control group (Figure 7). 

## 3. Discussion

FZD is widely used to treat the infections caused by Gram-positive and -negative bacteria in humans in some developing countries, including China, due to its better effects and lower cost [26]. FZD showed potential dose-dependent genotoxicity in a variety of test systems [9,11,27,28,29,30]. One previous study showed that oral administration of 40 mg/kg of FZD for 10 days could induce marked renal and hepatic toxicity in goat [31]. Gonzalez Borroto et al. demonstrated that FZD exposure at 20 μg/mL for 3 h could induce marked DNA damage in human lymphoblastic TK6 cells [6]. The present study aimed to investigate the protective effect of curcumin on FZD-induced DNA damage and apoptosis in human hepatocyte L02 cells. 

In the current study, FZD treatment at 10–60 μg/mL for 24 h significantly decreased the cell viability of L02 cells in a dose dependent manner. Curcumin pretreatment at the range of 1.25–5 μM relieved the decrease of cell viability (Figure 1), indicating that curcumin could protect L02 cells against FZD-induced cytotoxicity. ROS commonly consist of the superoxide radical anion, hydrogen peroxide (H_2_O_2_), and the hydroxyl radical [32]. An imbalance between the production of ROS and the antioxidant system defense will result in excessive ROS production, leading to damaging effects on lipids, proteins, and DNA, and ultimately cause cell death [33,34]. FZD is rapidly degraded in different tissues [2] and superoxide radical anion are produced during FZD metabolism [35]. MDA is used as a biomarker of oxidative stress to evaluate the degree of the peroxidation of membrane lipids [36]. In the present study, FZD exposure significantly increased the production of ROS and MDA content in L02 cells (Figure 2A,B). Besides, we also observed a decrease in GSH levels and the activity of the antioxidant enzymes SOD and CAT (Figure 2C–E). SOD can catalyze the dismutation of superoxide anion into oxygen and H_2_O_2_ [32]. CAT is a common enzyme and it can catalyze the decomposition of H_2_O_2_ to water and oxygen [32]. In addition, a previous study demonstrated that the rate of cellular H_2_O_2_ removal is partly dependent on GSH levels [37]. These antioxidant decreases might be due to FZD-induced production of free radicals which in turn can impair the antioxidant defense system, finally resulting in exacerbated oxidative stress. Studies have demonstrated that curcumin can directly interact with superoxide radical anion and H_2_O_2_ and inhibit oxidative stress even more so than vitamin E, which is an oxygen radical scavenger [38]. Previous studies have demonstrated that curcumin could effectively inhibit cytotoxicity and DNA injury induced by perfluorooctane sulfonate [39], quinocetone [40] and γ-radiation [41] by inhibiting ROS-mediated oxidative damage. Consistently, our study found that curcumin pre-treatment could not only inhibit the over-production of ROS induced by FZD, but also enhance the total antioxidant capacity in L02 cells, i.e., up-regulate the activities of SOD and CAT and GSH levels (Figure 2). This may be due to the antioxidant sparing action of curcumin. In addition, it has reported that curcumin can trigger the antioxidant response element (ARE) and induce some genes expression including SOD and CAT and GSH when the cells are under oxidative stress conditions [41], which also contributed to partly explain the phenomena observed in the present study (Figure 2). The comet assay is the most popular method for measuring various types of DNA damage, including oxidative damage inflicted by ROS [40]. Our results further showed that L02 cells exposed to FZD for 3 h suffered severe DNA damage, indicated by increased comet length, tail moment and olive tail moment, but no significantly cell death and apoptosis was observed. Curcumin remarkably prevented the FZD-induced DNA damage in L02 cells in a dose-dependent manner (Figure 6). Taken together, these data revealed that curcumin protected against FZD-induced cytotoxicity and DNA damage by inhibiting ROS formation and up-regulation of intracellular antioxidant levels. 

In the present study, FZD exposure led to an increase of apoptosis rates (Figure 5), characterized by cell shrinkage, chromatin condensation, nucleus condensation and the formation of apoptotic bodies [42]. Mitochondria are the most vulnerable targets of ROS and mitochondrial dysfunction may contribute to and cause cell death by trigging endogenous apoptotic cascade reactions [13,43]. As the data shows, FZD treatment caused a significant loss of membrane potential (Δψ_m_, Figure 3), an important characteristic indicative of mitochondrial dysfunction [44]. Disrupted Δψ_m_ may influence the opening of mitochondrial permeability transition pores (MPTPs), resulting in the release of cytochrome c (CytC) and cascading to the activation of caspase-9, -3 and apoptosome formation [45,46]. In the present study, curcumin treatment significantly attenuated the loss of Δψm, and decreased the mRNA expression levels of Bax, caspase-9 and -3, and increased the mRNA expression of Bcl-2, then attenuated FZD-induced apoptosis rates in L02 cells (Figure 3, Figure 4, and Figure 7). Bax and caspase-9 are also important markers of mitochondrial apoptotic pathways [45,46]. Bcl-2 is one of the anti-apoptotic Bcl-2 family proteins, which regulates the mitochondrial pathway [33]. Several studies showed that curcumin can block oxidative stress-mediated apoptosis via suppression of the mitochondrial apoptotic pathways, thus protecting against cisplatin, palmitate and 6-hydroxy-dopamine-induced cytotoxicity [47,48,49]. These data indicated that curcumin protected L02 cells against FZD-induced apoptosis via inhibiting mitochondrial pathways. 

It had demonstrated that the direct activation of Bax by p53 mediated mitochondrial membrane permeabilization, causing mitochondrial CytC release and caspase activation, which trigger apoptosis [46]. The nuclear translocation and transactivation of p53 were usually implicated in apoptotic cell death in response to oxidative stress [50]. A previous study had demonstrated that FZD could significantly increase the p53 protein expression in acute myeloid leukemia cells [3]. In the present study, FZD treatment significantly increased the mRNA level of p53 (Figure 7). Our previous studies had shown that p38 MAPK and GADD45a, a downstream gene of p53, participated in FZD-induced cell cycle arrest and apoptosis [51,52]. Another study demonstrated that curcumin protected against 6-hydroxydopamine-induced neurotoxicity through attenuation of p53-mediated apoptosis in the dopaminergic cell line SH-SY5Y [47]. Consistently, the present study showed curcumin treatment markedly decreased the p53 gene expression caused by FZD (Figure 7), indicating that the p53 pathway participates in the protective role of curcumin. The complete mechanism needs further in vitro and in vivo investigation.

**Figure 8:**
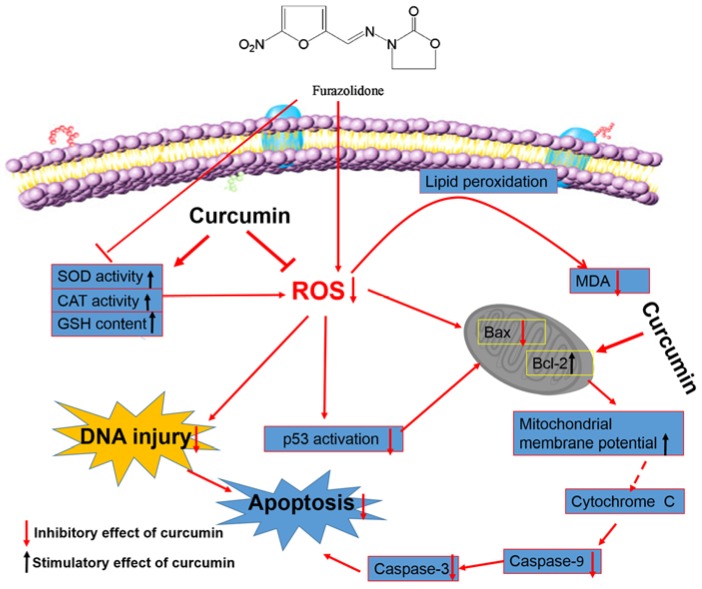
The schematic diagram of the possible mechanisms of curcumin protects against FZD-induced the cytotoxicity in L02 cells. There are several insights into the regulation mechanisms that contribute to the protective effect of curcumin on FZD induced cytotoxicity: (i) Inhibit the intracellular ROS formation and increase anti-oxidative enzyme activities to reduce DNA damage; (ii) Blockade of apoptosis by inhibiting mitochondrial pathway; (iii) Inactivation of p53 may contribute to the protective role of curcumin. Arrayed pink balls represent phospholipid bilayer of cell membrane.

In conclusion, this is the first study to demonstrate that curcumin could protect L02 cells against FZD-induced oxidative stress, DNA damage and cell apoptosis by inhibiting ROS production, enhancing the intracellular anti-oxidative ability and inhibiting the mitochondrial apoptotic pathway (Figure 8). Meanwhile, curcumin could effectively inhibit the p53 gene expression, which may partly contribute to the protective role of curcumin against FZD exposure-induced apoptosis (Figure 8). Importantly, the current study suggests that the combination of FZD with curcumin is a promising way to prevent the adverse effect of FZD in human or animals.

## 4. Materials and Methods

### 4.1. Chemical and Regents

Dulbecco’s Modified Eagle’s Medium (DMEM) and fetal bovine serum (FBS) were purchased from Invitrogen (Gibco, Grand Island, NY, USA). FZD, 3-(4,5-dimethyl-2-thiazolyl)-2,5-diphenyl-2*H*-tetrazolium bromide (MTT), Triton X-100 were purchased from Sigma-Aldrich (St. Louis, MO, USA). Curcumin (Aladdin Reagent Co., Ltd., Shanghai, China) was prepared as a 10 mM stock solution in dimethyl sulfoxide (DMSO, Sigma-Aldrich) and stored at −20 °C for standby. All other reagents were of analytical reagent grade.

### 4.2. Cell Culture

Human hepatocyte L02 cells were purchased from the Cell Bank of the Chinese Academy of Sciences (Shanghai, China). L02 cells were cultured in DMEM containing with 2% L-glutamine, 10% FBS, penicillin (100 units/mL, Gibco), and streptomycin (100 μg/mL, Gibco) in a humidified incubator at 37 °C with 5% CO_2_. The media was changed once per day.

### 4.3. Measurement of Cell Viability 

Cell viability was examined using MTT assay method according to the previous study with modifications [34]. In brief, L02 cells (1 × 10^4^ cells/well) were seeded into 96-well plate and incubated overnight. The cells were treated with curcumin at the final concentration of 1.25, 2.5 and 5 μM, respectively for 2 h, then washed twice with PBS and treated with FZD at 10–60 μg/mL or replaced with 0.2% DMSO (as the curcumin control). Cells in the negative control group were treated with 0.2% DMSO. After treatment for 24 h, the medium was discarded and incubated with 100 μL serum-free DMEM containing 10 μL MTT (5 mg/mL) for 4 h at 37 °C. Then, the medium was discarded and replaced with 100 μL DMSO. After incubation for 20 min at room temperature, the absorbance was read at 490 nm in a microplate reader (Molecular Devices, Sunnyvale, CA, USA).

### 4.4. Measurement of Intracellular ROS Production

The production of intracellular ROS was measured using the ROS-specific fluorescent dye 2,7-dichlorofluorescein diacetate (DCFH-DA) (Beyotime, Haimen, China). In brief, L02 cells were plated into 96-well plate and pretreated with curcumin at 1.25 and 2.5 and 5 μM at 37 °C for 2 h; then, the cells were washed twice with PBS and treated with FZD at 40 μg/mL or replaced with 0.2% DMSO (as the curcumin control). Cells in the negative control group were treated with 0.2% DMSO. After treatment for 24 h, L02 cells were washed three times with PBS, followed to add 200 μL DMEM containing 10 μM DCFH-DA and incubate for 30 min at 37 °C in the dark. After three washes with PBS, DCF fluorescence intensity was measured using a microplate fluorescence reader (excitation wavelength: 488 nm and emission wavelength: 530 nm) (Spectramax M3, Molecular Devices).

### 4.5. Measurement of the Activities of SOD, CAT and the Levels of MDA and GSH

The activities of SOD, CAT and the levels of MDA and GSH were measured using their specific assay kits and the methods were according to the manufacturer’s instructions (Nanjing Jiancheng Co., Ltd., Nanjing, China). In brief, L02 cells (2 × 10^5^ cells/well) were pre-treated with or without curcumin at 1.25, 2.5 and 5 μM for 2 h; then, the cells were washed twice with PBS and treated with FZD at 40 μg/mL or replaced with 0.2% DMSO (as the curcumin control). Cells in the negative control group were treated with 0.2% DMSO. After treatment for 24 h, L02 cells were washed with ice-cold PBS and lysed using the cell lysis buffer provided by the manufacturer. The collected lysates were centrifuged at 14,000 × *g* for 10 min at 4 °C. Then, the supernatants were collected to measure the activities of SOD and CAT and the levels of MDA and GSH. Protein contents were examined using a BCA™ protein assay kit (Beyotime). Values of the activities of SOD, CAT and the levels of MDA and GSH were corrected based on the protein contents.

### 4.6. Measurement of Δψ_m_

The changes of Δψ_m_ were determined using the Rhodamine 123 (Rh123) method, according to our previous study [53] with some modifications. In brief, L02 cells (2  ×  10^5^ cells/well) were pre-treated with curcumin at 1.25 and 2.5 and 5 μM at 37 °C for 2 h; then, the cells were washed twice with PBS and treated with FZD at 40 μg/mL or replaced with 0.2% DMSO (as the curcumin control). Cells in the negative control group were treated with 0.2% DMSO. After treatment for 24 h, L02 cells were incubated with 10 µg/mL Rh123 in the dark for 30  min at 37  °C. After washing twice with PBS, fluorescence image was observed using an inverted fluorescence microscope (Leica Microsystems, Wetzlar, Germany) and analyzed by the Image Pro Plus 5.0 software (Media Cybernetics, Inc., Silver Spring, MD, USA).

### 4.7. Measurement of Caspase-9 and -3 Activities

The activities of caspase-9 and -3 were determined using the Caspase-9 and -3 assay kits according to the manufacturer’s instructions (Beyotime), respectively. In brief, L02 cells were plated into 6-well plates at a density of 5  ×  10^5^ cells per well and pretreated with curcumin at 1.25, 2.5 and 5 μM at 37 °C for 2 h; then, the cells were washed with PBS twice and treated FZD at 40 μg/mL or replaced with 0.2% DMSO (as the curcumin control). Cells in the negative control group were treated with 0.2% DMSO. After treatment for 24 h, L02 cells were washed with ice-cold PBS and lysed using the cell lysis buffer provided by the manufacturer. The collected lysates were centrifuged at 14,000× *g* at 4 °C for 10 min. The supernatants were used to measure the activities of caspase-9 and -3. Protein concentrations were examined using a BCA™ protein assay kit (Beyotime). Values of the activities of caspase-9 and -3 were corrected based on the protein contents.

### 4.8. Measurement of Apoptosis

Cell apoptosis were examined using flow cytometric analysis and Hoechst 33342 staining. In brief, L02 cells (2  ×  10^5^ cells/well) were cultured on 6-well culture plates and pretreated with curcumin at 1.25, 2.5 and 5 μM at 37 °C for 2 h; then, the cells were washed with PBS twice and treated with FZD at 40 µg/mL for additional 24 h. Cells in the curcumin control group were treated with curcumin at 5 μM for 2 h, followed to wash with PBS twice and treat with 0.2% DMSO for additional 24 h. Cells in the negative control group were treated with 0.2% DMSO for 24 h. After treatment, the cells were harvested and detected using an annexin V-FITC apoptosis detection kit (Vazyme Biotech Co., Ltd., Nanjing, China) according with the protocol described in the previous study [14]. For Hoechst 33342 staining, the treated L02 cells were stained with 1 μg/mL Hoechst 33342 (Vigorous Biotechnology, Beijing, China) for 20 min in the dark, then observed under a fluorescence microscope (excitation wavelength at 340 nm and emission wavelength at 510 nm).

### 4.9. Measurement of DNA Damage by Alkaline Comet Assay

To detect cellular DNA damage as single-strand breaks, alkaline comet assay was performed using an Oxiselect Comet Assay® kit (Cell Biolabs, San Diego, CA, USA) according to the manufacturer’s instructions. In brief, L02 cells were pretreated with curcumin at 1.25, 2.5 and 5 μM for 2 h, then washed with PBS and exposed to FZD (40 μg/mL) or replaced with 0.2% DMSO (as the curcumin control) for 3 h. Cells in the negative control group were treated with 0.2% DMSO. To avoid artifacts resulting from necrotic and apoptotic cells, the cell suspensions (50 µL) were mixed with Hoechst 33342 (1 µg/mL) (Vigorous Biotechnology). After incubation in the dark for 30 min, necrosis and apoptosis were identified under a fluorescent microscope. In all groups, the cell viability was more than 90%. Then, the harvested cells were mixed with low melting point agarose and then transferred onto the CometSlide™ following solidification of cell-agarose mix. Cells were then incubated in lysis buffer (2.5 M NaCl, 100 mM EDTA, 10 mM Tris, pH 10 and 10% DMSO with 1% Triton X-100) in darkness at 4 °C for 1 h. After lysis, the slides were placed in alkaline solution (1mM Na_2_-EDTA and 300 mM NaOH, pH 13) for 20 min at room temperature to allow DNA unwinding. Then, electrophoresis was performed in alkaline solution for 30 min at 25 V. After electrophoresis, the slides were washed twice for 5  min each at 4  °C in a neutralizing buffer (0.4 M Tris, pH 7.5), dehydrated in 70% ethanol, stained with Vista Green DNA dye (provided with the kit). Images were observed using fluorescent microscopy at an excitation wavelength of 490 nm and emission wavelength of 530 nm (Leica, Omachi, Japan). At least 100 randomly selected cells (30 or 40 cells from each of the three replicated slides) were analyzed using Comet Assay Software Project casp-1.2.2 (University of Wroclaw, Wroclaw, Poland). The tail length is the length of the tail (in pixels); the tail DNA% is calculated as (tail DNA intensity/cell DNA intensity) × 100; the tail moment length is the length from the center of the head to the center of the tail; and the olive tail moment is calculated as the tail moment length  ×  tail DNA%.

### 4.10. Measurement of mRNA Expression of Apoptosis Factors by Quantitative Real-Time PCR (qRT-PCR)

L02 cells were pretreated with curcumin at 1.25, 2.5 and 5 μM at 37 °C for 2 h, followed to wash with PBS twice and treat with FZD or replaced with 0.2% DMSO (as the curcumin control). Cells in the negative control group were treated with 0.2% DMSO. After treatment for 24 h, total RNAs from cells were extracted using TRlzol^®^ reagent (Life Technologies, Grand Island, NY, USA) according to the manufacturer’s instructions. The cDNA was synthesized from 2 μg of total RNA using the Prime Script RT-PCR kit (Takara, Dalian, China). The PCR primers are listed in Table 1.

QRT-PCR was carried performed using an AB7500 real-time PCR instrument (Applied Biosystems, Foster City, CA, USA). After the amplification phase, a melting curve analysis was conducted to eliminate the possibility of non-specific amplification or primer dimer formation. All reactions were conducted in triplicate. The fold change in gene expression was calculated using 2^−ΔΔCt^ after normalizing to the expression level of β-actin.

### 4.11. Statistical Analysis

Data were shown as mean ± standard deviation from at least three independent experiments performed in duplicate or triplicate. A one-way analysis of variance, followed by a Fisher’s least significant difference (LSD) test, was employed to compare any two means when the variance was homogeneous; otherwise, Dunnett’s T3 test was used (SPSS Inc., Chicago, IL, USA). A *p* < 0.05 was considered significant difference.

## Figures and Tables

**Table 1:** The primer sequences for β-actin and other target genes of apoptosis factors.

Gene	Primer Sequences (5′–3′)	Product Size (bp)
Caspase-9	Forward: 5′-gaggttctcagaccggaaacac-3′ Reverse: 5′-catttcccctcaaactctcaaga-3′	90
Caspase-3	Forward: 5′-gcgaatcaatggactctggaat-3′ Reverse: 5′-aggttgctgcatcgacatctg-3′	270
Bax	Forward: 5′-gatgcgtccaccaagaagct-3′ Reverse: 5′-cggccccagttgaagttg-3′	169
Bcl-2	Forward: 5′-gcggagttcacagctctatac-3′ Reverse: 5′-aaaaggcccctacagttacca-3′	136
p53	Forward: 5′-ccctcctcagcatcttatcc-3′ Reverse: 5′-gcacaaacacgcacctcaa-3′	260
β-actin	Forward: 5′-gggaaatcgtgcgtgac-3′ Reverse: 5′-ttgccaatggtgatgacctg-3′	138
